# Evaluation of postoperative satisfaction with rhinoseptoplasty in patients with symptoms of body dysmorphic disorder

**DOI:** 10.1016/j.bjorl.2020.07.013

**Published:** 2020-09-12

**Authors:** Luísi Rabaioli, Paula de Oliveira Oppermann, Natália Paseto Pilati, Cássia Feijó Gomes Klein, Bárbara Luiza Bernardi, Raphaella Migliavacca, Michelle Lavinsky-Wolff

**Affiliations:** a Universidade Federal do Rio Grande do Sul (UFRGS), Programa de Pós-Graduação em Medicina, Ciências Cirúrgicas, Porto Alegre, RS, Brazil; b Hospital de Clínicas Porto Alegre (HCPA), Departamento de Otorrinolaringologia, Porto Alegre, RS, Brazil; c Universidade Federal do Rio Grande do Sul (UFRGS), Faculdade de Medicina, Porto Alegre, RS, Brazil

**Keywords:** Body dysmorphic disorders, Rhinoplasty, Surgery, plastic

## Abstract

**Introduction:**

The prevalence of body dysmorphic disorder among candidates for plastic surgery may vary from 6% to 54%. Some studies report discrete benefits with the surgical results, while others show symptomatic exacerbation. Some authors even affirm that body dysmorphic disorder would be a surgical contraindication, against others who suggest satisfactory results.

**Objective:**

To describe the prevalence of body dysmorphic disorder in rhinoseptoplasty candidates and to compare outcomes among patients with and without body dysmorphic disorder symptoms.

**Methods:**

Cohort study. Individuals ≥ 16 years, candidates for aesthetic and/or functional rhinoseptoplasty were recruited at a university hospital in Brazil. The prevalence of body dysmorphic disorder was assessed through the Body Dysmorphic Disorder Examination (BDDE) and the patients divided into groups: no symptoms of body dysmorphic disorder, mild-moderate and severe symptoms. The specific quality of life outcomes, Nasal Obstruction Symptom Evaluation (NOSE) and Rhinoplasty Outcome Evaluation (ROE) were evaluated before and after 90 and 180 days of the procedure.

**Results:**

131 individuals were included, 59.5% female. The prevalence of preoperative symptoms of body dysmorphic disorder was 38%. There was a reduction in the symptoms of body dysmorphic disorder in the preoperative body dysmorphic disorder examination versus 3 and 6 months in all groups (78.94 ± 2.46 vs. 33.63 ± 6.41 and 35.51 ± 5.92, respectively, *p* < 0.002). Among patients with severe body dysmorphic disorder symptoms, rhinoplasty outcome evaluation ranged from 21.24 ± 3.88 to 58.59 ± 5.83 at 3 months and 52.02 ± 5.41 at 6 months postoperatively (*p* < 0.001); while NOSE from 71 ± 8.47 to 36.11 ± 12.10 at 6 months postoperatively (*p* < 0.01).

**Conclusion:**

The prevalence of body dysmorphic disorder symptoms in our sample was high. Rhinoseptoplasty was associated with an improvement in quality of life outcomes related to nasal function and aesthetic outcome in all groups, irrespective of the presence and intensity of body dysmorphic disorder symptoms. Rhinoseptoplasty in body dysmorphic disorder symptomatic patients was also associated with a reduction in postoperative body dysmorphic disorder symptoms, even in severe cases.

## Introduction

Rhinoseptoplasty is a surgical procedure frequently performed in otolaryngology and facial plastic surgery, aiming to alter the shape or appearance of the nose, preserving, or improving its functionality and patency to the airflow.^1^ The demand for aesthetic surgeries has risen in an extremely significant way for the past years. According to data published by the International Society of Aesthetic Plastic Surgery (ISAPS), Brazil was the leading country in the number of aesthetic surgical procedures performed in the year 2018. When analyzing rhinoplasty alone, Brazil was the country that performed the procedure the most in 2018, responsible for11.8% of the world total (more than 80 thousand surgeries).^2^

For some people, the pursuit of bodily perfection can be confused with happiness and fulfillment, causing great frustrations when not attained. It is well known that most people have some degree of dissatisfaction with some feature of their appearance. However, when one takes into account the emphasis given to beauty by the current culture, it is a fact that the risk for the development of a disorder is high. Thus, people who seek to constantly change aspects of their physical appearance and are always dissatisfied with the results of aesthetic treatments and surgeries are highly likely to have a condition described as Body Dysmorphic Disorder (BDD).^3^

The BDD is characterized by extreme dissatisfaction with the body image caused by the cognitive distortion of the self-image, that is, the affected individuals are worried about one or more minimal or even nonexistent defects in their physical appearance, which they believe to appear ugly, abnormal or deformed. These concerns are intrusive, unwanted, time-consuming (occurring, on average, from 3 to 8 h a day) and are often difficult to resist or control.^3^

Prevalence is estimated between 1% and 3% in the general population,^4^ but among candidates for plastic surgery, it may vary from 6% to 54% according to literature.^4^, ^5^, ^6^, ^7^, ^8^, ^9^, ^10^, ^11^, ^12^ This large variability in the prevalence rates of body dysmorphic disorder in a cosmetic setting can be attributed to the difference in the interpretation and application of diagnostic criteria and also to socio-cultural discrepancies. Although the severity of the symptoms is quite variable, these distorted concerns cause clinically significant distress and impairment in personal, social, and professional functioning, leading to depression, anxiety, suicidal ideation, and avoidant social behavior with impaired quality of life.^13^, ^14^ Most of these people undergo cosmetic treatments, but there seems to be a poor response to such treatments and sometimes worsening of symptoms.^5^

Due to the high prevalence of body dysmorphic disorder in the candidate population for aesthetic surgeries, the surgeon must be able to evaluate psychological motivations and expectations from surgeryl in the preoperative period, aiming to differentiate the patients with true indications for surgical treatment from those affected by psychiatric disorders, capable of interfering in the body self-perception, avoiding unnecessary procedures with unsatisfactory surgical results.^10^, ^15^ The paucity of evidence in the literature on the subject justifies a study to determine the prevalence of BDD in rhinoseptoplasty candidates, as well as the impact on postoperative outcomes.^6^

Studies that have already been carried out present inconsistent conclusions: some report discrete benefits with the surgical results of rhinoplasty, while others show symptomatic BDD exacerbation in the post-operative period. Some authors even affirm that BDD would be a surgical contraindication, against others who suggest satisfactory results.^7^, ^10^, ^16^, ^17^, ^18^ The vast majority of clinical reports and retrospective studies have shown that patients with BDD show a low degree of satisfaction and postoperative deterioration of the symptoms of the disorder. The worst results are usually described in patients undergoing multiple surgeries.^11^, ^18^, ^19^, ^20^

The objectives of the present study are to describe the prevalence of BDD in a population of candidates for aesthetic and/or functional rhinoseptoplasty, and to compare outcomes of aesthetic and functional satisfaction among patients with and without BDD symptoms.

## Methods

### Design and participants

The present study was conducted at the outpatient clinic of Facial Plastic Surgery of the Otolaryngology Service of a tertiary care university hospital in the in the south of Brazil, from March 2016 to September 2018. It is a cohort study which included patients of both sexes, older than 16 years, candidates for aesthetic, functional or associated rhinoseptoplasty. Exclusion criteria were: (1) Additional concomitant procedures, such as functional endoscopic sinus surgery, adenoidectomy, blepharoplasty or otoplasty; (2) Previous diagnosis of BDD with or without treatment; (3) Unrealistic surgical motivations; (4) Objectives unattainable by technical difficulties.

The study was approved by the Research Ethics Committee under the registry #15-0520. The Informed Consent Form was obtained from each patient before inclusion in the study.

### Sample size

To estimate the prevalence of positive screening for BDD in the study population, the 9% frequency reported in the study of Felix et al. was taken as a base.^20^ The sample calculation was performed through Winpepi version 11.65, adopting a 95% confidence level, resulting in an estimated sample of 126 individuals for preoperative analysis. For the detection of 20 points of difference in the ROE scale in the comparative analysis between the pre- and postoperative groups among patients with and without BDD, using as reference the scores obtained by Lavinsky-Wolff et al. in 2012, the sample was calculated in 22 individuals, 11 of them in each group, considering a level of significance of 5% and a power of 80%.^21^

### Data collection and questionnaires

Between March 2016 and September 2018, 131 septorhinoplasty candidates who met the inclusion criteria were consecutively included in the study. All of them were submitted to a clinical interview and to a questionnaire on demographic and baseline characteristics during the preoperative evaluation. The evaluation instruments described below were applied by trained and blinded interviewers before surgery, as well as 90 and 180 days after surgery. Surgeries were performed by a single surgeon with the assistance of medical residents. Patients who had nasal obstruction associated with aesthetic complaints were evaluated for the presence of septal deviations and turbinate hypertrophy and, when necessary, surgical interventions were performed for functional improvement. Surgeons did not have access to the results of the questionnaires before surgery or during follow-up. The surgical technique was not standardized to meet the specific needs of each patient.

1 BDDE (Body Dysmorphic Disorder Examination): Scale for screening for symptoms of BDD validated for Portuguese based on 34 questions regarding the concern with appearance, negative self-image, self-consciousness, avoidance of activities, body camouflage, defect checking, and psychological symptoms. The items are rated on a scale from 0 to 6, with 0 indicating the absence of negative body image symptoms in the last 4 weeks. Scores from 1 to 6 represent the frequency (days) or intensity (mild to severe) of the symptoms. The maximum score is 168 and a cutoff point above 66 indicates a greater degree of dissatisfaction with appearance and is generally associated with the diagnosis of BDD. The classification of the physical deformity perceived by the patient was performed by the interviewers during the preoperative evaluation. Severe cases were those that met DSM-IV criterion B (the concern causes clinically significant distress or impairment in social, occupational or other important areas of functioning), that is, those with BDDE scores ≥ 4 in items 23, 24 and (25 or 26).^3^, ^22^
2 Rhinoplasty Outcome Evaluation (ROE): quality of life questionnaire validated in Portuguese for evaluation of results in patients submitted to rhinoplasty. It consists of six questions that involve three domains of quality of life: physical, mental/emotional, and social. According to this scale, the score ranges from 0 to 100; the higher score indicates the greater degree of satisfaction.^23^, ^24^
3 Nasal Obstruction Symptom Evaluation Scale (NOSE): questionnaire with 5 items about quality of life, specifically to evaluate nasal obstruction in clinical trials, already validated in Portuguese (NOSE-p). A score of 0 means that there are no problems related to nasal obstruction while a score of 100 means the most serious problem possible with nasal obstruction.^25^, ^26^, ^27^

### Statistical methods

Analysis was performed using SPSS software version 25. Continuous variables were described as mean and standard deviation and categorical variables as percentages. The value of *p* ≤ 0.05 indicates statistical significance. Analysis of variance (ANOVA) was used for comparison of means between groups; Student’s *t*-test was used for variables of normal distribution and Chi-square of Pearson for the other variables. For intragroup comparisons, pre and postoperative, the Generalized Estimating Equation (GEE) test was used which evaluates all completed questionnaires at all visits.^28^

## Results

Participants were divided into three groups according to their preoperative score in the BDDE: patients without symptoms of BDD (total score < 66), patients with mild-moderate BDD symptoms (total score ≥ 66) and patients with severe BDD symptoms (total score ≥ 66 and scores ≥ 4 on items 23, 24 and 25 or 26). The demographic data for these groups are described in Table 1.

**Table 1:** Socio-demographic characteristics according to the severity of BDD symptoms in the preoperative score (n = 131).

Characteristics	Without symptoms (n = 81)	Mild-moderate symptoms (n = 39)	Severe symptoms (n = 11)
Age, mean (SD), years	37.57 (14.36)	34.33 (12.86)	36.09 (13.75)
Sex			
Female	43 (53.1%)	29 (74.4%)	6 (54.5%)
Education (years)			
Up to 8 years	31 (38.3%)	10 (25.6%)	4 (36.4%)
9–11 years	39 (48.1%)	18 (46.2%)	4 (36.4%)
12 years or more	11 (13.6%)	11 (28.2%)	3 (27.3%)
Comorbidities	35 (43.2%)	16 (41%)	2 (18.2%)
Previous nasal surgery	13 (16.3%)	3 (7.7%)	1 (9.1%)
History of nasal fracture	29 (35.8%)	12 (30.8%)	6 (54.5%)
Surgery main concern			
Functional	24 (29.6%)	4 (10.3%)	0
Aesthetic	4 (4.9%)	1 (2.6%)	1 (9.1%)
Both	53 (65.4%)	34 (87.2%)	10 (90.9%)

A total of 131 patients was included, with a mean age of 36.33 ± 14.12 years, 59.5% female. The prevalence of positive screening for BDD in the preoperative evaluation was 38% (n = 50). In patients without symptoms of BDD, the mean age was 37 ± 14 years; 34 ± 12 in those with mild to moderate symptoms and 36 ± 13 in patients with severe symptoms, ranging from 17 to 71 years. No statistically significant association was found between presence or severity of BDD symptoms and age, sex, presence of comorbidities, educational level, history of nasal fracture, previous nasal surgery. However, there was a higher prevalence of exclusive functional rhinoplasty among patients without symptoms of BDD (29%), whereas a greater search for aesthetic rhinoplasty was found in the group with severe symptoms of BDD (9.1%) (*p* = 0.03).

The scores obtained in the BDDE, NOSE and ROE questionnaires were also compared among groups at the preoperative visit (Table 2). The mean BDDE score of all participants was 78.94 ± 2.46, 32 ± 2.14 for patients without symptoms of BDD, 89 ± 2.83 for mild-moderate symptoms and 114 ± 6.48 for severe symptoms (*p* < 0.001). Quality of life specific to nasal obstruction (NOSE scores) were comparable among groups, however, there was a difference in aesthetic satisfaction (ROE scores) among patients without symptoms of BDD versus mild to moderate and without symptoms versus severe symptoms (*p* = 0.01 and <0.001, respectively).

**Table 2:** Preoperative score of BDDE, NOSE and ROE questionnaires according to the severity of the symptoms of BDD (n = 131).

	BDDE mean ± SE^a^	ROE mean ± SE^b^	NOSE mean ± SE
Without symptoms (n = 81)	32.90 (2.14)	37.75 (16.68)	68.64 (22.62)
Mild-moderate symptoms (n = 39)	89.02 (2.83)	29.27 (14.44)	68.33 (27.0)
Severe symptoms (n = 11)	114.90 (6.48)	21.96 (12.51)	64.54 (34.31)

SE, Standard Error.

a ANOVA (Analysis of Variance) for comparison of means (*p* < 0.01).

b ANOVA − groups without symptoms vs. mild-moderate (*p*  = 0.01), group without symptoms vs. severe (*p* < 0.01).

Preoperatively, BDDE scores were 30.39 ± 30.77 for functional rhinoseptoplasty patients versus 62.49 ± 34.05 for associated functional and aesthetic ones and 71.83 ± 41.30 for the exclusively aesthetic patients (*p* < 0,02 and *p* < 0.001, respectively).

**Figure 1:**
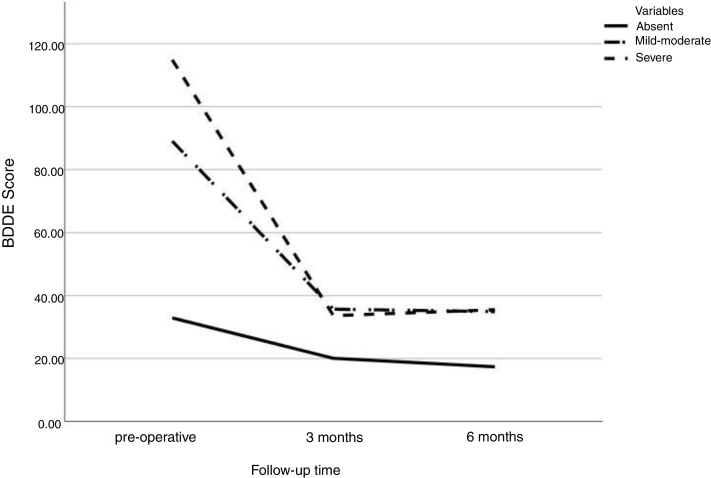
Variation of preoperative BDDE scores, 3 months, and 6 months after surgery, according to the severity of BDD symptoms in the preoperative phase. (SE, Standard Error). BDDE scores, mean ± SE: group without symptoms BDD in the preoperative phase 32.9 ± 2.14; 3 months 20.06 ± 3.19; 6 months 17.36 ± 2.93; mild-moderate preoperative symptoms 89.02 ± 2.83; 3 months 35.68 ± 5.77; 6 months 34.91 ± 7.10; severe preoperative symptoms 114.90 ± 6,48; 3 months 45.16 ± 18.06; 6 months 54.25 ± 16.03.
2 NOSE: NOSE scale mean score was 50.91 ± 33.88, ranging from 0 to 100. Although there was a significant difference between the pre- and postoperative values, the variation of the scores was similar between patients without BDD, with mild-moderate BDD and severe BDD (*p* < 0.001) (Table 3).

1 BDDE: There was a significant difference in the BDDE score between groups in the preoperative phase: group without symptoms of BDD 32.9 ± 2.14, with mild-moderate symptoms 89.02 ± 2.83 and with severe symptoms 114.90 ± 6.48 (*p* = 0.001). During follow-up, the difference in BDDE score between preoperative visits versus 3 months postoperative and preoperative versus 6 postoperative months was significant for all groups (*p* = 0.002), however no difference was found between 3 and 6 postoperative months (Fig. 1).

**Table 3:** Comparison of preoperative NOSE scores and 6 months after surgery+, according to the severity of preoperative BDD symptoms.

NOSE	Preoperative, mean ± SE^a^ (n = 130)	6 months postoperative, mean ± SE (n = 75)	Total, mean ± SE (n = 130)
Without symptoms^a^	68.61 (2.49)	19.92 (3.87)	29.51 (1.65)
Mild-moderate symptoms^a^	66.41 (4.60)	22.67 (4.81)	44.54 (3.29)
Severe symptoms^a^	71.0 (8.47)	36.11 (12.10)	53.55 (9.59)

SE, Standard Error.

a Generalized Estimating Equation (GEE), *p* < 0.01 for all groups during follow-up.
3 ROE: The mean score was 56.75 ± 26.56 points, ranging from 4.17 to 100. When evaluating each visit, we observed a significant variation between the groups: without symptoms versus severe BDD (77.09 vs. 58.59 and 79.56 vs. 52.02, *p* = 0.004 and 0.05 respectively) and mild-moderate versus severe (74.01 vs. 58.59 and 71.25 vs. 52.02, *p* < 0.001 and 0.009 respectively) after 3 and 6 months of postoperative follow-up, respectively.

The comparison of the intragroup scores during followup showed a significant difference (*p* < 0.001) between the preoperative score versus 3 months (without symptoms 37.7 vs. 77.09, mild-moderate symptoms 29.9 vs. 74.01 and severe symptoms 21.2 vs. 58.5) and preoperative versus 6 months for all groups (without symptoms 37.7 vs. 79.5, mild-moderate symptoms 29.9 vs. 71.2 and severe symptoms 21.2 vs. 52.02), without variation between 3 and 6 postoperative months (Table 4).

**Table 4:** Comparison of preoperative ROE scores, 3 months, and 6 months after surgery, according to the severity of preoperative BDD symptoms. **(**SE = standard error).

ROE	Pre-operative, mean ± SE (n = 130)	3 months mean ± SE (n = 91)^†^^a^	6 months mean ± SE (n = 75)^b^	Total, mean ± SE (n = 296)
Without symptoms^c^	37.77 (1.84)	77.09 (2.20)	79.56 (2.12)	64.81 (1.32)
Mild-moderate symptoms^c^	29.91 (2.18)	74.01 (3.58)	71.25 (3.59)	58.39 (2.35)
Severe symptoms^c^	21.24 (3.88)	58.59 (5.33)	52.02 (5.41)	43.95 (3.52)

SE, Standard Error.

a Generalized Estimating Equations (GEE), *p*  = 0.004 and *p* = 0.05, between groups without symptoms vs. severe and mild-moderate vs. severe, respectively.

b Generalized Estimating Equations (GEE), *p* < 0.001 and 0.009 for groups without symptoms vs. severe and mild-moderate vs. severe, respectively.

c Generalized Estimating Equations (GEE), *p* < 0.001 for difference between preoperative scores vs. 3 months and preoperative vs. 6 months in all groups.

## Discussion

Nasal appearance is among the main preoccupations of the patients affected by BDD, leading them to frequently search for septorhinoplasty. Most studies report a prevalence ranging from 6% to 54%.^4^, ^5^, ^6^, ^7^, ^8^, ^9^, ^10^, ^11^, ^12^ The prevalence in our study agrees with those previously performed in population candidates for aesthetic surgeries, revealing a score of BDD symptoms in 38% of the patients, and 8% presented severe BDD scores. Although Brazil is one of the world leaders in aesthetic procedures, the literature is scarce in national data on rhinoseptoplasty and BDD. Felix et al. in 2013 presented data from 31 Brazilian patients, exclusively females, with occurrence of BDD symptoms in 59% and 21% of them had scores for severe BDD symptoms.^20^ This rate is high when compared to the general population (about 2%) and, therefore, it becomes important to investigate and detail this disorder and its impact on symptoms and outcomes.

The option of including patients over 16 years old is justified by facial development and nasal growth, since after that age there is already greater safety for rhinoplasty.^29^ 22 patients between 16 and 20 years old were included (10 men and 12 women), representing 17% of the sample. The prevalence of BDD in this age group is estimated between 1.7% to 3.2%, similar to the prevalence of the general population (2.5%).^30^, ^31^

The use of a self-completed screening questionnaire aimed to approximate reality and allow independence to the surgeon, allowing identification of suggestive symptoms and need for additional research and treatment. The BDDE has no diagnostic purposes: we cannot say that the patient with positive screening actually has the disorder, since its definitive diagnosis requires the evaluation of presented deformities, as well as a skilled professional in mental health.

Another crucial finding of our study was the significant decrease in the scores of BDD symptoms related to the preoperative period (in the preoperative comparison at 3 months follow-up, a reduction of 12.84 points for the group without symptoms of BDD, 53.34 points for the group with mild-moderate symptoms and 60.65 for the group with severe symptoms). Although most of the literature suggests unsatisfactory results and worsening of the BDD with rhinoseptoplasty, our results indicated that severity of BDD is not associated with surgical satisfaction, since there was also a reduction in the BDD symptom scores after the surgery of the surgical procedure.^11^, ^12^, ^14^, ^18^

These results agree with the previous Brazilian study, which showed complete remission of BDD symptoms in 81% of patients with mild-moderate scores and postoperative satisfaction in 90% after 1 year of surgery.^11^, ^32^ The total BDDE scores in our sample are comparable to those presented by De Brito et al.^32^ both preoperatively (78.94 ± 2.4 vs. 107.2 ± 18.2, respectively) and in follow-up (33.63 ± 6.41 in 3 months vs. 52.8 ± 24.3 in 12 months, respectively), although they did not include male patients.

Among the characteristics evaluated in the preoperative period, only the purpose of the surgery was associated with the severity of BDD. We found higher BDDE scores in patients with aesthetic complaints (isolated or associated with functional ones), as described by Picavet et al.^12^

In addition, the preoperative satisfaction with the nasal appearance measured by the ROE score was significantly lower in the group with scores for BDD symptoms. Also, there was a significant postoperative increase in nasal satisfaction, measured by the ROE questionnaire, in all groups (in preoperative comparison with 3 months follow-up, an increase of 39.32 points for the group without symptoms, 44.1 points among patients with mild-moderate symptoms and 30.78 points for patients with severe symptoms).

Moreover, during followup, we observed a significant reduction in nasal obstruction scores (NOSE) for all groups with no difference during 3- and 6 month visits. Our findings suggest that even patients with BDD can realize an improved quality of life related to nasal obstruction after rhinoplasty, independent of the severity of BDD symptoms. Thus, having a positive screening for BDD does not necessarily mean worse postoperative outcomes.

One of the main limitations of the study is the short-term followup. However, since quality of life outcomes were similar on 3- and 6 months assessments, one can suppose that the results should be the stable over time, which should be confirmed in future studies. Also, it is important to highlight that although we had satisfactory follow-up over time, our sample remained higher than the value initially calculated to detect differences among groups.

## Conclusion

Rhinoseptoplasty was associated with an improvement in quality of life outcomes related to nasal function and aesthetics, irrespective of the presence and intensity of BDD symptoms. In BDD symptomatic patients, rhinoseptoplasty was also associated with a reduction in postoperative BDD symptoms, even in severe cases.

## Funding

Fundo de Incentivo à Pesquisa e Eventos (FIPE) from Hospital de Clínicas de Porto Alegre and Conselho Nacional de Desenvolvimento Científico e Tecnológico (10.13039/501100003593CNPq).

## Conflicts of interest

The authors declare no conflicts of interest.
